# A Rare Encounter With Unusual Metastasis: Metastatic Right Ventricular Tumor Secondary to Cervical Cancer

**DOI:** 10.7759/cureus.62530

**Published:** 2024-06-17

**Authors:** Ekenedilichukwu N Nnadi, Nazima Khatun, Sabu John

**Affiliations:** 1 Internal Medicine, State University of New York Downstate Medical Center, Brooklyn, USA; 2 Cardiology, Kings County Hospital Center, Brooklyn, USA

**Keywords:** cardiac imaging-mri, cardio-oncology, transthoracic echocardiography (tte), multimodality cardiac imaging, cervical cancer, cardiac metastasis

## Abstract

Metastasis of cervical cancer to the heart is rare. Cervical carcinoma typically spreads to the lungs, liver, bones, and lymph nodes via hematogenous, lymphatic, transvenous, or direct extension. Cardiac metastasis from cervical carcinoma is uncommon and portends a dismal prognosis, with mean survival under six months post-diagnosis. A high index of suspicion and multimodal imaging is imperative for prompt diagnosis and improved outcomes in these patients. Here, we report a rare case where a 41-year-old African American female with stage IIIB cervical squamous cell carcinoma (SCC) presented with exertional dyspnea and chest pain concerning pulmonary embolism (PE). Computed tomography angiography showed no PE but revealed a right ventricular (RV) mass and diffuse pulmonary nodules. Echocardiography suggested an RV tumor versus a thrombus. Cardiac magnetic resonance imaging demonstrated a large RV infiltrative mass favoring metastasis over thrombus. A biopsy of one of the pulmonary nodules confirmed metastatic SCC. Despite treatment, the prognosis was poor.

## Introduction

Cervical cancer is the second most common malignant tumor observed globally in females [1]. Metastasis is a common complication in advanced stages, but metastasis to the heart is an exceedingly rare and clinically significant event. The incidence of metastatic cardiac tumors, based on autopsy studies, is reported to be around 1.2% [2]. However, this may be an underestimation as these tumors are frequently asymptomatic, found incidentally through imaging performed for unrelated reasons, or can go disguised until during autopsy. Hypothetically, any cancer can metastasize to the heart, with the usual sources being breast, esophagus, and lung malignancies. Infra-diaphragmatic organ malignancies rarely metastasize to the heart, such as in the case of cervical cancer [3].

Here, an unusual case of cervical cancer metastasizing to the right ventricle (RV) of the heart as a large mass is presented, underscoring the importance of detecting atypical metastases. Documenting this rare cardiac spread contributes insights to guide clinicians in similar scenarios and inform diagnosis, treatment decisions, and disease progression awareness. A multidisciplinary approach between oncology, cardiology, and radiology is emphasized for optimal management.

## Case presentation

A 41-year-old African American woman presented to the emergency room with progressively worsening shortness of breath for two weeks with associated pleuritic chest pain. On arrival, the patient showed no fever, exhibited a heart rate of 117 beats per minute, had a blood pressure of 111/72 mmHg, and maintained an oxygen saturation of 99% while breathing ambient air. On examination, the anterior chest wall was tender to touch, but no other abnormalities were noted.

Her past medical history included diabetes mellitus and the International Federation of Gynecology and Obstetrics stage IIIB cervical squamous cell carcinoma (SCC) of the cervix. The patient’s chemotherapy/radiotherapy history included three cycles of carboplatin/paclitaxel and two cycles of gemcitabine/cisplatin. Due to complications of severe thrombocytopenia, she was switched to weekly cisplatin and Tandem and Ovoid high-dose-rate brachytherapy.

Initial labs were significant for hemoglobin of 7.3 g/dL (with a baseline ~9 g/dL) with a normal mean corpuscular value of 88.2 fL, and a white blood count of 3.91 K/µL. The comprehensive metabolic panel was relatively within the patient’s normal limits. Detailed laboratory results are listed in Table 1.

**Table 1:** Initial laboratory test results.

Laboratory tests	Results	Reference range
Sodium	141	136–145 mmol/L
Potassium	4.1	3.5–4.8 mmol/L
Magnesium	1.77	1.6–2.6 mg/dL
Phosphorus	3.4	2.3–4.7 mg/dL
Chloride	102	98–107 mmol/L
Carbon dioxide	27	22–29 mmol/L
Glucose	109	77–100 mg/dL
Blood urea nitrogen	27	7–20 mg/dL
Creatinine	0.86	0.6–1.1 mg/dL
Glomerular filtration rate	>60	≥60 mL/minute/1.73 m^2^
Total protein	7.2	6.7–8.6 g/dL
Total bilirubin	0.2	0.2–1.2 mg/dL
Aspartate aminotransferase	11	5–34 U/L
Alanine transaminase	7	0–37 U/L
Alkaline phosphatase	54	40–150 U/L
Calcium	9.7	8.4–10.4 mg/dL
Troponin T	0.010	<0.010 ng/mL
B-type natriuretic peptide	672	<100 pg/mL
White blood cell count	3.91	3.80–10.80 K/µL
Hemoglobin	7.8	12–16 g/dL
Hematocrit	26.3	34–45%
Mean corpuscular volume	88.2	80–99 fL
Platelet count	158	150–400 K/µL
Venous blood pH	7.37	7.31–7.41
Venous blood partial pressure of carbon dioxide	48	30–50 mmHg
Venous blood bicarbonate	28	23–28 mmol/L
Lactate	0.8	0.5–1.6 mmol/L

**Figure 1:**
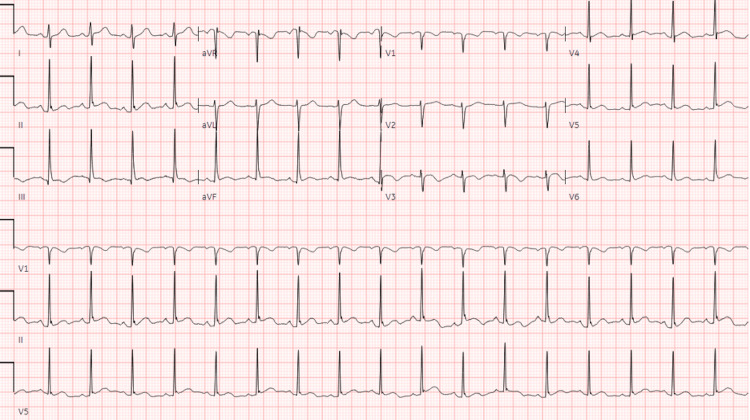
An electrocardiography demonstrating sinus tachycardia.

An initial electrocardiogram exhibited sinus tachycardia at a heart rate of 106 beats per minute (Figure 1).

**Figure 2:**
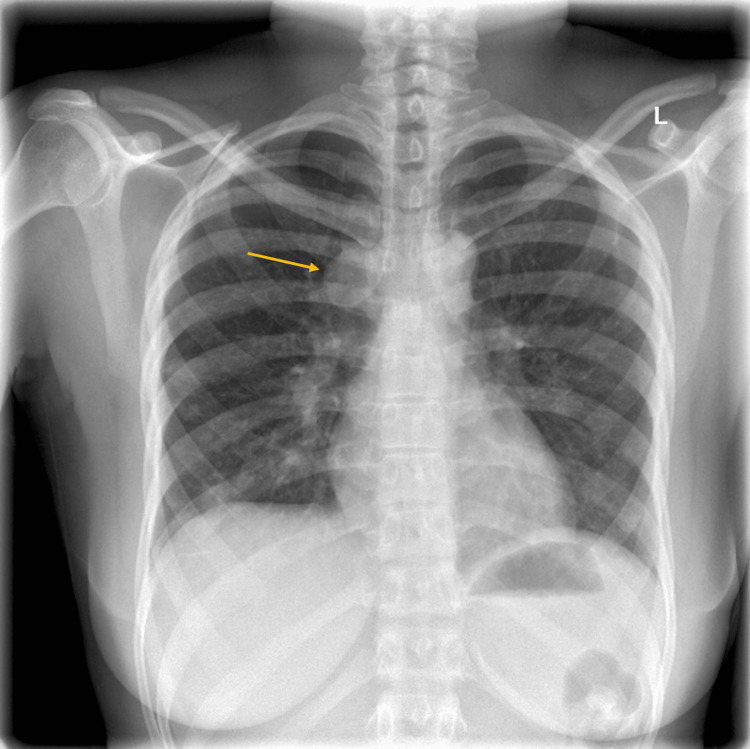
A posterolateral view of a chest X-ray. The image demonstrates an oval-shaped high-density focus measuring 2.9 × 2 cm and projecting over the medial aspect of the left upper lung overlying the fifth and sixth posterior ribs (yellow arrow).

Chest X-ray showed an oval-shaped, high-density focus measuring 2.9 × 2 cm projecting over the medical aspect of the left upper lung overlying the fifth and sixth posterior ribs (Figure 2).

**Figure 3:**
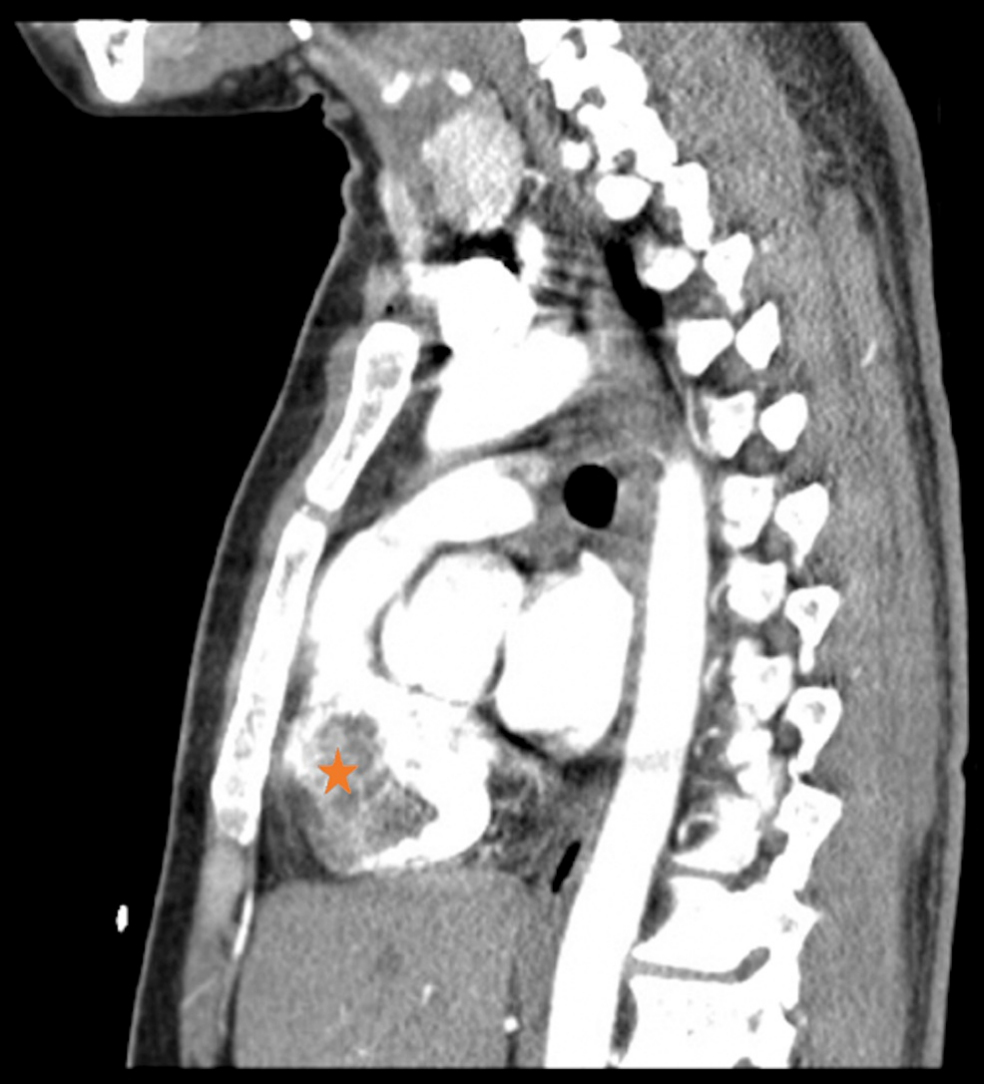
Computed tomography of the chest with contrast (sagittal view) showing a hypoenhancing defect within the right ventricle (asterisk).

Computed tomography angiography (CTA) showed no signs of pulmonary embolism in the segmental pulmonary arteries. However, it did reveal the emergence of multiple diffuse bilateral pulmonary nodules, along with mediastinal and right hilar lymphadenopathy. Additionally, an incidental discovery was made of a hypoenhancing defect in the RV (Figure 3).

**Figure 4:**
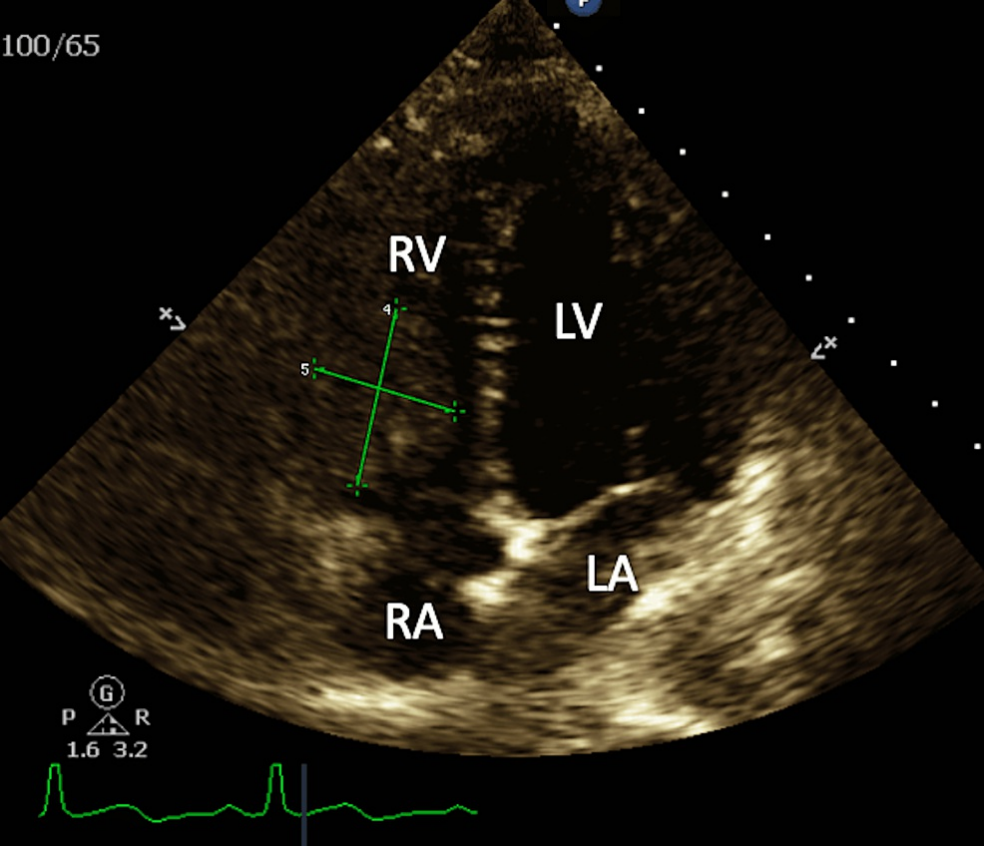
A four-chamber view of transthoracic echocardiography showing a right ventricular mass. RV: right ventricle; LV: left ventricle; RA: right atrium; LA: left atrium

The patient was admitted to the hospital for further investigation of CTA findings concerning thrombus versus mass in the RV and subsequently started on a treatment dose of enoxaparin. The following hospital day, an echocardiogram was done and revealed normal left ventricular systolic function with an ejection fraction of 60% and a large mass attached to the lateral wall of the RV just above the lateral tricuspid annulus approximately 3.3 cm × 2.0 cm in size (Figure 4).

**Figure 5:**
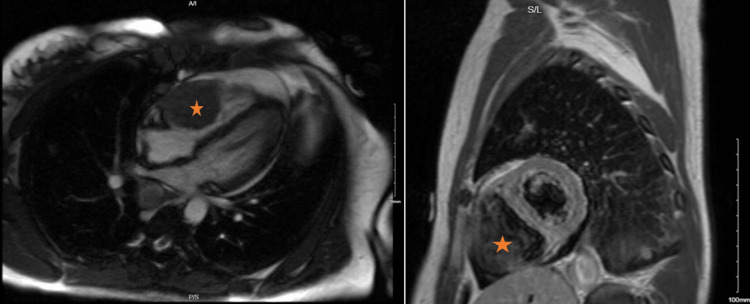
Cardiac magnetic resonance imaging. The image shows a large irregularly shaped mass measuring up to 4.0 × 3.3 cm and extending from the mid-RV free wall to the base of the RV and partially protruding into the RA during systole with imaging characteristics of a tumor (asterisk). RV: right ventricle; RA: right atrium

On hospital day five, cardiac magnetic resonance imaging (cMRI) confirmed a large mass infiltrative irregularly shaped mass measuring 40 cm × 33 cm extending from the mid-RV free wall to the base of the RV partially protruding through the tricuspid valve in the right atrium during systole with imaging characteristics of a tumor (Figure 5).

Enoxaparin was discontinued. During interdisciplinary rounds, given the higher risk of RV mass biopsy, the decision was made to biopsy the right lower lobe lung nodule which revealed metastatic SCC with p16, p40, and PD-L1 positive, combined positive score 3. Given the poor prognosis and the unlikely chance of achieving a curative resection or eliminating all signs of the disease, the patient was considered unsuitable for RV mass resection. Following limited response to several rounds of chemotherapy and radiotherapy, the patient opted to undergo palliative treatment. The patient remained hemodynamically stable throughout the hospital stay. Consequently, the patient was discharged to initiate outpatient therapy involving tisotumab vedotin and pembrolizumab, alongside supportive palliative care.

## Discussion

Metastases of cervical cancer typically involve lymph nodes, liver, bones, liver, and lungs. Metastases of cervical cancer to the heart, although an extremely rare phenomenon, usually occur to the right heart, as in our case, with the incidence of cardiac metastasis of uterine cervix tumors shown in autopsy studies to be between 3% and 4% with many cases diagnosed postmortem [4,5]. The rarity of cardiac metastasis can be attributed to several factors, such as the constant contraction of the heart muscle, the unique metabolic characteristics of striated cardiac muscle, the swift blood flow through the heart, and the direction of lymph flow away from the heart [6]. In this case, with the involvement of the right heart, it is presumed that hematogenous spread occurred through the cervical venous plexus, along with tumor cell filtration in the lungs [7-9].

Multimodal imaging studies are important for the early detection of cardiac metastasis, including transthoracic and transesophageal echocardiography, CTA of the heart, cMRI, and positron emission tomography-computed tomography [8]. Echocardiography is an invaluable non-invasive and cost-effective image modality that is preferred for detecting and differentiating intracavitary cardiac tumors. A limitation of echocardiography is the high risk of misidentifying masses in the right ventricle as thrombosis, as seen in our case, which can lead to treatment delays [10]. MRI remains the modality of choice in the non-invasive evaluation of intrinsic myocardial abnormalities [8].

Management for cardiac metastasis of cervical cancer has not been standardized due to the poor prognosis [4]. The mean survival time from diagnosis is four months, with the literature search demonstrating the longest survival after the diagnosis of cardiac metastasis from cervical cancer of 13 months [11,12]. Optimal treatment should be tailored on an individual basis, involving a dedicated multidisciplinary team. While opinions may vary, it is generally agreed that to extend survival and enhance the quality of life, a multimodal management approach incorporating surgery, chemotherapy, radiotherapy, and biological therapy is beneficial [9]. As in this current case, patient care focused on palliative care due to the poor response to chemotherapy.

## Conclusions

This case illustrates an uncommon yet clinically impactful occurrence of cervical cancer metastasizing to the heart. Cardiac involvement from cervical primary lesions is distinctly rare. This case highlights the need for prompt consideration of metastatic cardiac disease in cervical cancer patients manifesting with atypical signs or symptoms. A multimodality diagnostic approach is essential to facilitate timely recognition. While management options for this entity are limited, early diagnosis can guide appropriate individually tailored therapy to optimize quality of life. Overall, maintaining a high degree of suspicion is critical, as early identification confers the best opportunity to alter the disease course in this rare and deadly phenomenon.
